# Supplementary material for: Fusion surface structure, function, and dynamics of gamete fusogen HAP2
Source: eLife. 2018 Oct 3;7:e39772. doi: 10.7554/eLife.39772 (PMC6170185; doi:10.7554/eLife.39772)
Supplement: Supplementary file 1. — (a) The numbers in parentheses refer to the highest resolution shell. (b) Rmerge = Σh Σi |Ii(h) - < I(h)> | / ΣhΣi Ii(h), where Ii(h) and <I(h)>are the ith and mean measurement of the intensity of reflection h. (c) Pearson’s correlation coefficient between average intensities of random half-datasets for unique reflection (Karplus and Diederichs, 2012). (d) Rfactor = Σh||Fobs (h)|-|Fcalc (h)|| / Σh|Fobs (h)|, where Fobs (h) and F calc (h) are the observed and calculated structure factors, respectively. No I/σ(I) cutoff was applied. e Calculated with MolProbity (Davis et al., 2007). [file elife-39772-supp1.docx]

**Supplementary File 1. Crystal data collection and refinement statistics.**

|  |  |
| --- | --- |
| **Data collection statistics** |  |
| Space group | C2 |
| α, β, *γ*, ° | 90, 123.8, 90 |
| Unit cell (a, b, c), Å | 198.2, 117.6, 115.2 |
| Resolution range (Å) | 50.0-2.60(2.67-2.60) ^a^ |
| Completeness (%) | 97.3 (85.8) ^a^ |
| Number unique reflections | 66,071 (4,346) ^a^ |
| Redundancy | 4.7 (3.3) ^a^ |
| R_merge_ (%) ^b^ | 9.5 (355.9) ^a^ |
| I/σ(I) | 9.4 (0.33) ^a^ |
| CC_½_ (%)^c^  Wavelength (Å) | 99.9 (16.0) ^a^  1.071951 |
| **Refinement statistics** |  |
| R_work_ (%)^d^ | 24.0 |
| R_free_ (%)  Bond RMSD (Å)  Angle RMSD (°) | 28.1  0.006 |
|  | 1.07 |
| Twin law | HKL,  -1/2H-1/2K+L, -1/2H-1/2K-L, 1/2H-1/2K,  -1/2H+1/2K+L, 1/2H-1/2K+L, 1/2H+1/2K |
| Twin fractions | 0.345,0.338,0.317 |
| Ramachandran plot  (Favored/allowed/outlier) ^e^  Molprobity percentiles  (Clash/Geometry) ^e^ | 95.0/5.0/0  100/100 |
| PDB code | 6DBS |

a The numbers in parentheses refer to the highest resolution shell.

b Rmerge = Σh Σi |Ii(h) -<I(h)> | / ΣhΣi Ii(h), where Ii(h) and <I(h)> are the i^th^ and mean measurement of the intensity of reflection h.

c Pearson’s correlation coefficient between average intensities of random half-datasets for unique reflection ^40^.

d Rfactor = Σh||Fobs (h)|-|Fcalc (h)|| / Σh|Fobs (h)|, where Fobs (h) and F calc (h) are the observed and calculated structure factors, respectively. No I/σ(I) cutoff was applied.

e Calculated with MolProbity ^41^.
